# Thalidezine, a novel AMPK activator, eliminates apoptosis-resistant cancer cells through energy-mediated autophagic cell death

**DOI:** 10.18632/oncotarget.15616

**Published:** 2017-02-22

**Authors:** Betty Yuen Kwan Law, Flora Gordillo-Martínez, Yuan Qing Qu, Ni Zhang, Su Wei Xu, Paolo Saul Coghi, Simon Wing Fai Mok, Jianru Guo, Wei Zhang, Elaine Lai Han Leung, Xing Xing Fan, An Guo Wu, Wai Kit Chan, Xiao Jun Yao, Jing Rong Wang, Liang Liu, Vincent Kam Wai Wong

**Affiliations:** ^1^ Macau Institute for Applied Research in Medicine and Health, State Key Laboratory of Quality Research in Chinese Medicine, Macau University of Science and Technology, Macau, China

**Keywords:** thalidezine, AMPK activator, autophagy, autophagic cell death, apoptosis-resistant cancer

## Abstract

Cancers illustrating resistance towards apoptosis is one of the main factors causing clinical failure of conventional chemotherapy. Innovative therapeutic methods which can overcome the non-apoptotic phenotype are needed. The AMP-activated protein kinase (AMPK) is the central regulator of cellular energy homeostasis, metabolism, and autophagy. Our previous study showed that the identified natural AMPK activator is able to overcome apoptosis-resistant cancer via autophagic cell death. Therefore, AMPK is an ideal pharmaceutical target for chemoresistant cancers. Here, we unravelled that the bisbenzylisoquinoline alkaloid thalidezine is a novel direct AMPK activator by using biolayer interferometry analysis and AMPK kinase assays. The quantification of autophagic EGFP-LC3 puncta demonstrated that thalidezine increased autophagic flux in HeLa cancer cells. In addition, metabolic stress assay confirmed that thalidezine altered the energy status of our cellular model. Remarkably, thalidezine-induced autophagic cell death in HeLa or apoptosis-resistant DLD-1 *BAX-BAK* DKO cancer cells was abolished by addition of autophagy inhibitor (3-MA) and AMPK inhibitor (compound C). The mechanistic role of autophagic cell death in resistant cancer cells was further supported through the genetic removal of autophagic gene7 (Atg7). Overall, thalidezine is a novel AMPK activator which has great potential to be further developed into a safe and effective intervention for apoptosis- or multidrug-resistant cancers.

## INTRODUCTION

The AMP-activated protein kinase (AMPK), a serine/threonine kinase, is a heterotrimeric complex containing one catalytic α subunit and regulatory β and γ subunits. As a major sensor of cellular energy homeostasis, AMPK is sensitive to intracellular ATP levels. AMP or ADP can directly bind to the regulatory γ subunits of AMPK, leading to a conformational change that promotes its activation [1]. Full AMPK activation requires specific phosphorylation of the α subunit at Thr172 by upstream kinases [2, 3]. Once activated, AMPK restores cellular energy levels by promoting catabolic and inhibiting anabolic processes. AMPK directly phosphorylates a number of downstream targets including ACC, ULK (protein kinases that initiate autophagy), and mTORC1 (mammalian target of rapamycin complex 1), which act as mediator/effectors to metabolism, cell growth, and autophagy [1, 4–6]. Autophagy (‘self-eating') is highly regulated by the autophagic genes (*Atg*) in eukaryotic cells. The process engulfs regions of cytoplasm, protein aggregates, and damaged organelles into double-membrane autophagosomes, which fuse with lysosomes for further degradation and cellular recycling [5–7]. Thus far, more than 37 *Atgs* have been discovered, for example, *Atg1*, *Atg6*, and *Atg12*, which are responsible for the proper functioning of the autophagic machinery [8]. Amongst the different *Atg*, *Atg*7 is a unique E1 enzyme that facilitates the membrane recruitment of autophagic proteins (ATG) critical to the autophagosome formation [8]. Autophagy may exert a multifactorial influence on the initiation and progression of cancer, as well as on the effectiveness of the associated therapeutic interventions. In apoptosis-resistant cancer cells, autophagy may facilitate chemotherapeutic or radiation-induced cytotoxicity through autophagy-associated cell-death pathways [9–12].

The role of AMPK as a possible metabolic tumour suppressor and a target for cancer prevention and treatment has received increasing interest [13–15]. In part, due to the epidemiological studies of metformin, the most widely prescribed type 2 diabetes drug, has been shown to activate AMPK, and reduce the incidence of various cancer models [16]. Recently, several AMPK activators including 5-aminoimidazole-4-carboxamide-1-b-d-ribofuranoside (AICAR), glycolysis inhibitor (2-DG), Abbott A769662 or mitochondrial inhibitors (biguanides or thiazolidinediones) [17] have been developed. Natural compounds such as resveratrol, a polyphenol, can activate AMPK and inhibit the mitochondrial ATPase, which protect against metabolic disease [18].

Owing to the low toxicity and effectiveness in nature, natural products have been studied and used worldwide as potential chemopreventive agents. Chinese herbal medicines (CHM) provide great potential to isolate new drugs for clinical applications [19]. For example, Thalidezine is one of the alkaloids isolated and identified from *Thalictrum glandulosissimum* in 1967 [20]. The medicinal plant is an ancient perennial herb of China with a history of folkloric use in the therapy of acute infections, acute enteritis and dysentery, conjunctivitis, pyogenic dermatitis, and acute laryngopharyngitis [21, 22]. One of the main components of *T. glandulosissimum*, hernandezine, was found to possess anti-cancer properties in both mice and *in vitro* models. Thalidezine and isothalidezine isolated from this plant also possessed inhibitory effects on mouse leukemia L1210 cells [23]. However, detail regarding the functions or mechanisms of thalidezine are still elusive.

In our current study, we have identified a novel AMPK activator, thalidezine, isolated from the *T. fendleri* [20], which was able to induce autophagic cell death in a panel of apoptosis-resistant cells, *via* the AMPK-mTOR and Atg 7 dependent mechanism.

## RESULTS

### Thalidezine directly binds and activates AMPK

**Figure 1:**
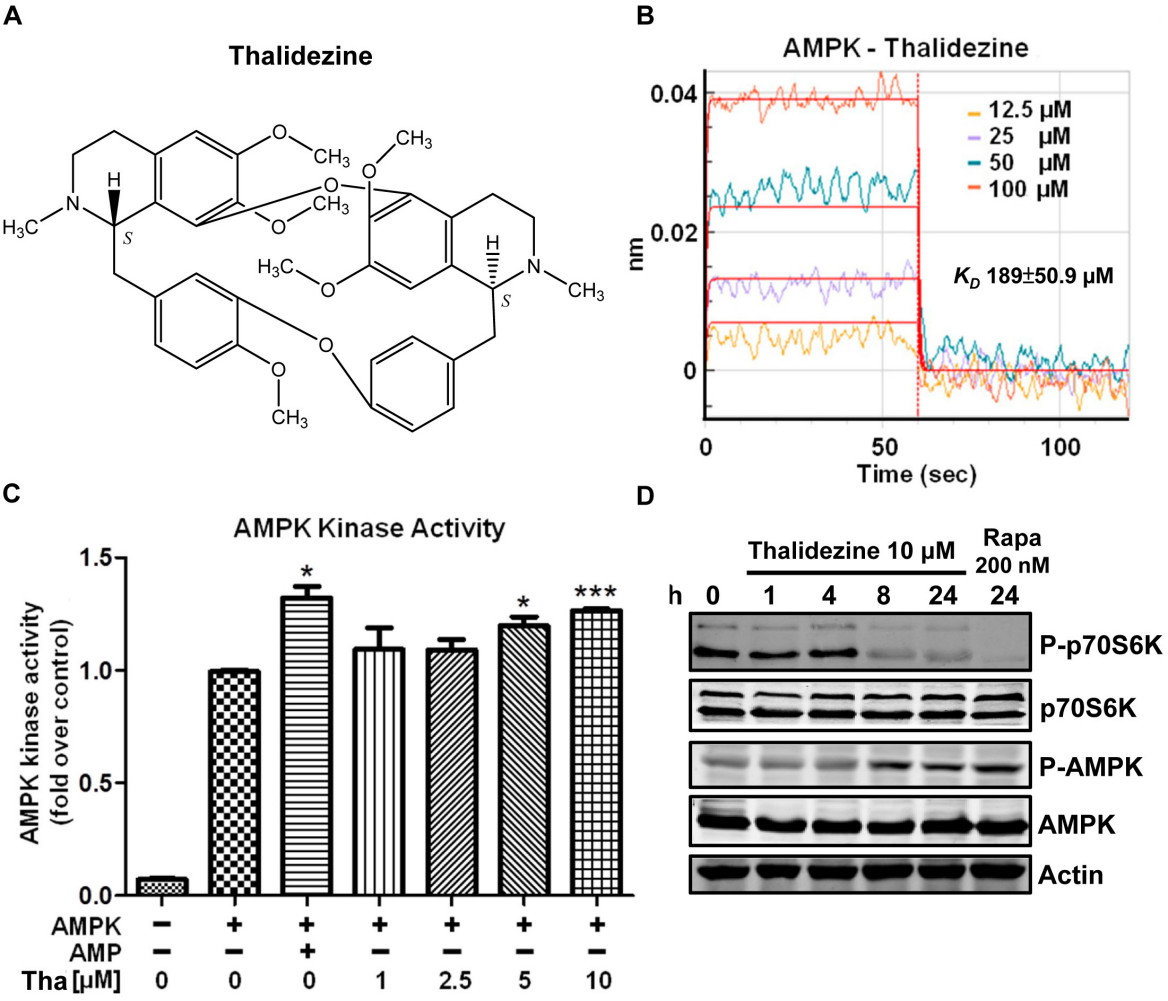
Thalidezine binds and activates AMPK *in vitro* **(A)** Chemical structure of thalidezine. **(B)** Kinetic analysis of the interaction between AMPK and thalidezine by BLI. The Ni-NTA biosensor tips coated with HIS-tagged AMPK were dipped in increasing concentrations of thalidezine (12.5, 25, 50, and 100 μM) to measure binding affinity of thalidezine to AMPK (*K_on_* 1.17±0.231×104 Ms^−1^) and subsequently moved to wells containing buffer to measure dissociation rates (*K_off_* 2.22±0.407 s^−1^). The affinity constant was calculated as the ratio of the *K_off_* to the *K_on_* (*K_D_* 189±50.9 μM). **(C)** Thalidezine directly activates AMPK kinase. AMPK protein was incubated without (control) or with increasing concentrations of thalidezine (Tha) (1, 2.5, 5, and 10 μM) or AMP (10 μM, positive control) for 20 min. *, *P* ≤ 0.05; **, *P* ≤ 0.01; ***, *P* ≤ 0.001. **(D)** Thalidezine activates the AMPK-mTOR signaling pathway. HeLa cells were treated with 10 μM of thalidezine for 0-24 h, rapamycin (Rapa, 200 nM) was used as the positive control. Immunoblots indicated p-AMPK, total AMPK, p-p70S6K, total p70S6K, and β-actin detection. Uncropped blots images were shown in Supplementary Figure 4A. Data were representative of three to five independent experiments.

AMPK has attracted widespread interest as a potential therapeutic target for cancer. A number of direct AMPK activators have been reported [17, 24]. Consistent with our previous works, we proposed a new class of compound exhibiting direct activation of AMPK, the bisbenzylisoquinoline alkaloid compounds such as liensinine, isoliensinine, dauricine, cepharanthine and hernandezine [25, 26]. Here, thalidezine (Figure 1A), a structural isomer of hernandezine C_39_H_44_N_2_O_7_ (Supplementary Figure 1A), shows different structural conformation (Supplementary Figure 1B), having six different possible conformers compare with three for hernandezine [27]. First, to investigate if thalidezine directly binds and activates the widely expressed α1β1γ1 isoform of mammalian AMPK, we determined the *in vitro* binding kinetics by bio-layer interferometry (BLI) and the AMPK activity. Thalidezine was found to bind directly to AMPK protein, the affinity equilibrium constant revealed a medium-high affinity with *K_D_* value of 189 μM (Figure 1B). Thalidezine showed higher affinity binding compare to hernandenzine (Supplementary Figure 1C). The interaction between thalidezine and AMPK promoted its kinase activation in a dose-response manner (Figure 1C). The effectiveness of thalidezine was then determined by Western blot for AMPK phosphorylation in HeLa cells. Immunoblot results indicated an increase in AMPK phosphorylation accompanied by a reduction in phosphorylated p70S6K, a downstream target of mTOR, in response to thalidezine after eight hours of treatment (Figure 1D). These findings clearly indicate that thalidezine directly binds to and activates AMPK.

### Thalidezine shows specific cytotoxic effect towards a panel of cancer cells

**Figure 2:**
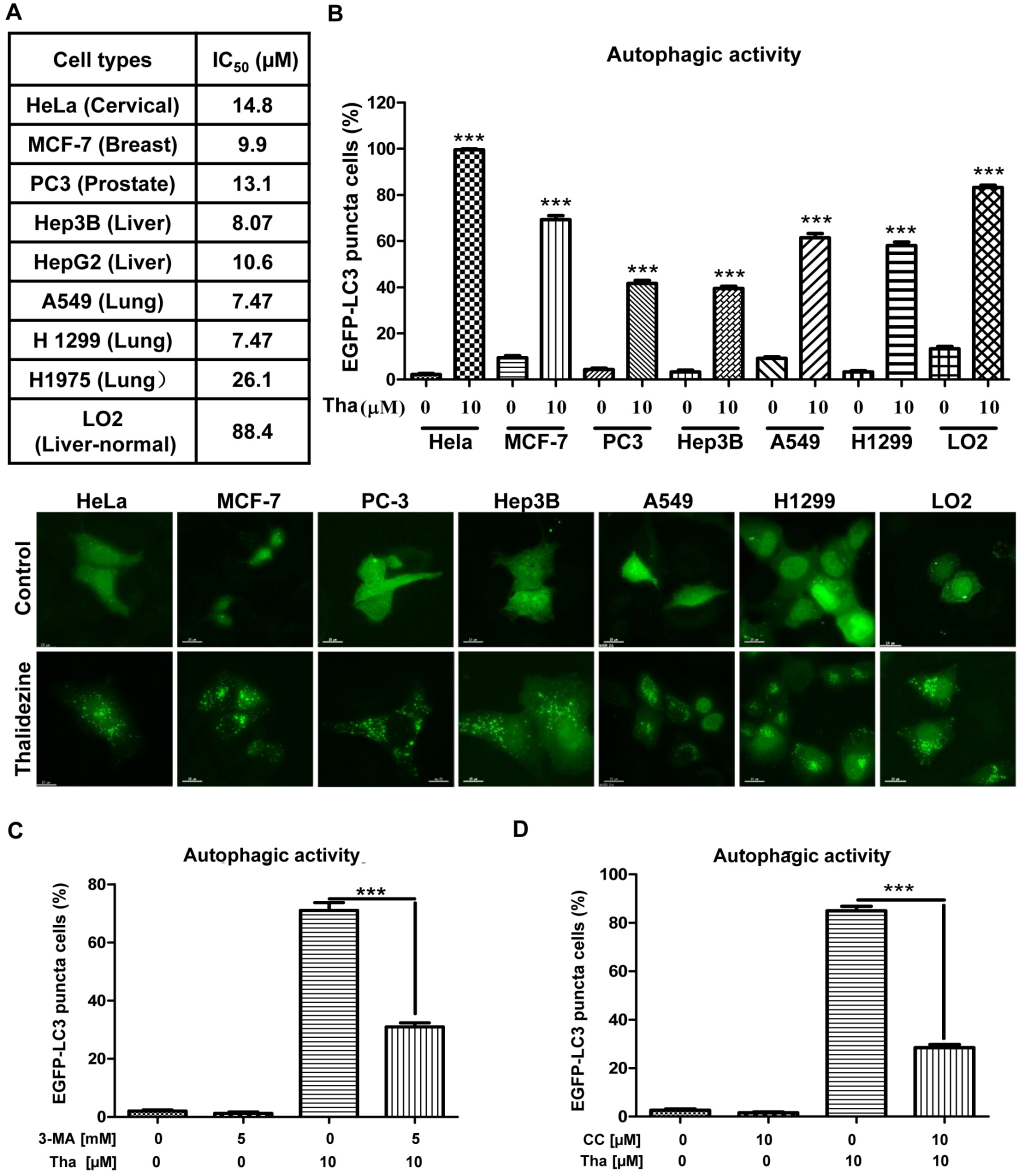
Thalidezine shows specific cancer cells cytotoxicity and induces autophagy **(A)** Thalidezine exhibits specific cell cytotoxicity toward a panel of cancer and normal cells. Mean IC_50_ values were shown in the table. **(B)** Thalidezine induces EGFP-LC3 puncta formation in HeLa, MCF-7, PC3, Hep3B, A549, H1299 cancer cells and LO2 normal hepatocytes (lower panel). Cells were treated with DMSO or 10 μM of thalidezine for 24 h. Percentage of cells with EGFP-LC3 puncta formation quantification was indicated as upper panel. **(C** & **D)** Autophagic inhibitor 3-MA and AMPK inhibitor CC respectively abolish thalidezine-mediated autophagy. HeLa cells transiently transfected with EGFP-LC3 plasmid were treated with DMSO or 10 μM thalidezine (Tha) with or without 3-MA (5 mM), and with or without CC (5 μM) for 4h. Bar charts quantification of cells with increased EGFP-LC3 puncta formation were shown. Representative fluorescence microscopy images are shown in Supplementary Figure 2B & 2C. ***, *P* ≤ 0.001. Data were mean value ± S.D of three independent experiments.

To evaluate the potential anti-cancer effect of thalidezine, a panel of cancer cells from different origins, including HeLa, A549, MCF-7, PC3, HepG2, Hep3B, H1299, and H1975 were utilized in the cytotoxicity test, whereas the LO2 normal human hepatocytes cell line was used as normal control cells. The mean IC_50_ values of thalidezine indicated a potent cytotoxic effect towards all these cancer cells, especially on A549 lung cancer (7.47 μM), H1299 lung cancer (7.47 μM), Hep3B liver cancer (8.07 μM), MCF-7 breast cancer (9.9 μM), and HepG2 liver cancer (10.6 μM). Interestingly, thalidezine exhibited relatively low cytotoxicity towards LO2 normal liver hepatocytes (mean IC_50_, 88.4 μM) suggesting that thalidezine is an effective anticancer agent with considerably less toxicity towards normal cells (Figure 2A).

### Thalidezine induces autophagy activity via AMPK activation in cancer cells

Although a number of natural alkaloid compounds have been identified as autophagy inducers for treatment of cancers [25, 28], none of them were validated with direct protein targets. To investigate whether thalidezine exhibits an autophagy effect on cells through AMPK activation, we utilized various types of cancer cells from different origins including HeLa, MCF-7, PC-3, Hep3B, A549, and H1299, as well as LO2 normal human hepatocytes for detection of autophagic EGFP-LC3 puncta. As shown in Figure 2B, 10 μM of thalidezine could induce EGFP-LC3 puncta formation among these cancer and normal cells, indicating that the autophagic effect of thalidezine is not cell-type specific. Interestingly, by quantitation of cells with EGFP-LC3 puncta formation, thalidezine demonstrated various level of autophagic potency among these cancer cells, whereas HeLa cancer cells were the most susceptible ones in response to thalidezine-mediated autophagy (Figure 2B). Therefore HeLa cells were selected as a model for autophagy studies. In addition, the formation of LC3-II puncta was verified by immunofluorescence staining against endogenous LC3-II (Supplementary Figure 2A). Alternatively, thalidezine-induced autophagic effect was further validated with 3-methyladenine, which is the PI3K inhibitor commonly used to suppress autophagy [29–31]. The addition of 3-MA significantly blocked the thalidezine-mediated autophagy, as shown by the decreased percentage of cells with EGFP-LC3 puncta (Figure 2C and Supplementary Figure 2B). Furthermore, pharmacological blockade of the AMPK signalling pathway by compound C (CC) impaired the autophagy-inducing effect of thalidezine (Figure 2D and Supplementary Figure 2C), suggesting the thalidezine-induced autophagy requires AMPK activation. Collectively, our results confirm the autophagy-inducing activity of thalidezine through the AMPK activation.

### Thalidezine induces autophagic flux in HeLa cells

**Figure 3:**
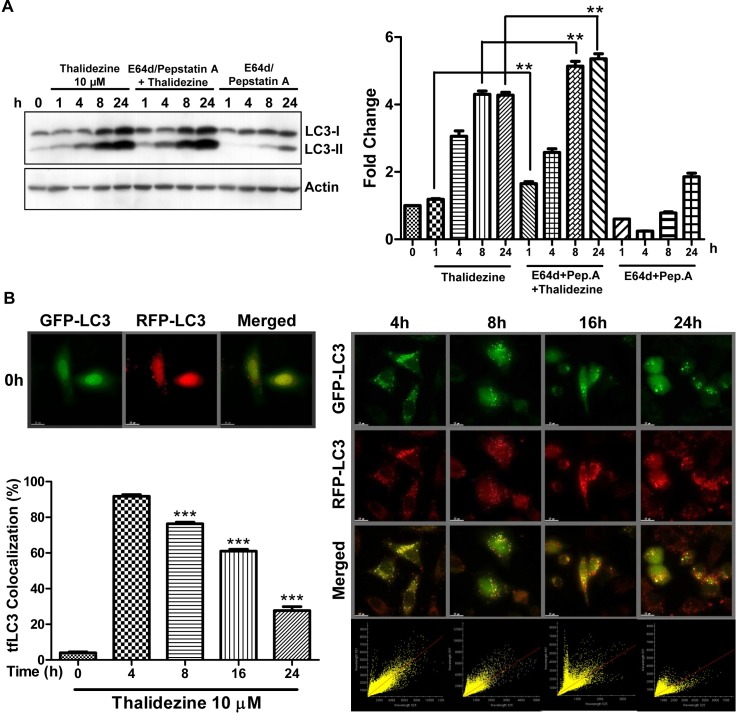
Thalidezine induces autophagic flux in HeLa cells **(A)** Thalidezine induces LC3-II conversion in the presence of lysosomal inhibitors. HeLa cells were treated with 10 μM of thalidezine in the presence or absence of 10 μg/mL lysosomal protease inhibitors (E64d and pepstatin A) for 24 h. Immunoblot for LC3-I, LC3-II, and β-actin detection (left). LC3 conversion was expressed as fold change relative to the DMSO-treated negative control (right). Uncropped blots images are shown in Supplementary Figure 4C. **(B)** tfLC3 fluorescence detection pattern of thalidezine. HeLa cells were transfected with mRFP-GFP-LC3 plasmids and treated with 10 μM of thalidezine (Tha) for 0-24 h. Representative micrographs of cells with GFP-LC3 puncta (green channel), mRFP puncta (red channel), and merge images are shown (upper-left and right panel). Scale bar = 15 μm, 60X. Each correlation plot was derived from the field shown in the fluorescence image (lower-right panel). Histogram represented the quantification of the percentage of colocalization between mRFP and GFP signal (lower-left panel). **, *P* ≤ 0.01; ***, *P* ≤ 0.001. Data were mean value ± S.D of at least three independent experiments.

Since the increase in autophagosomes by measuring EGFP-LC3 puncta formation might be a result of either the induction of autophagy flux or a failure in fusion of autophagosomes and lysosomes, the conversion of soluble LC3-I to lipid-bound LC3-II was measured in the presence of lysosomal protease inhibitors (E64d and pepstatin A) or bafilomycin A by western blot [30–32]. Obviously, thalidezine markedly increased the rate of LC3-II formation in the presence of the inhibitors when compared with the use of either inhibitors or thalidezine treatment alone (Figure 3A and Supplementary Figure 2D). This result suggested that thalidezine induced autophagic activity through enhanced autophagy flux and autophagosome formation. Alternatively, we continued to trace autophagy flux using mRFP-GFP tandem fluorescent-tagged LC3 (tfLC3) method, which detect the different pattern of GFP-LC3 and tfLC3 based on the different stabilities of GFP and mRFP under certain pH conditions [33]. As the acidic condition of the lysosome quenches the green fluorescence (GFP) signal but not the red fluorescence (mRFP) signal, the LC3 fusion construct with red and green fluorescence proteins is therefore commonly adopted for the determination of autolysosomes (red puncta) and autophagosomes (merged images, yellow puncta) [34]. Our results demonstrated a time-dependent reduction in the percentage of cells with mRFP-GFP co-localization after thalidezine treatment (Figure 3B), confirming the induction of autophagy flux by this alkaloid compound.

### Thalidezine suppresses energy metabolism

**Figure 4:**
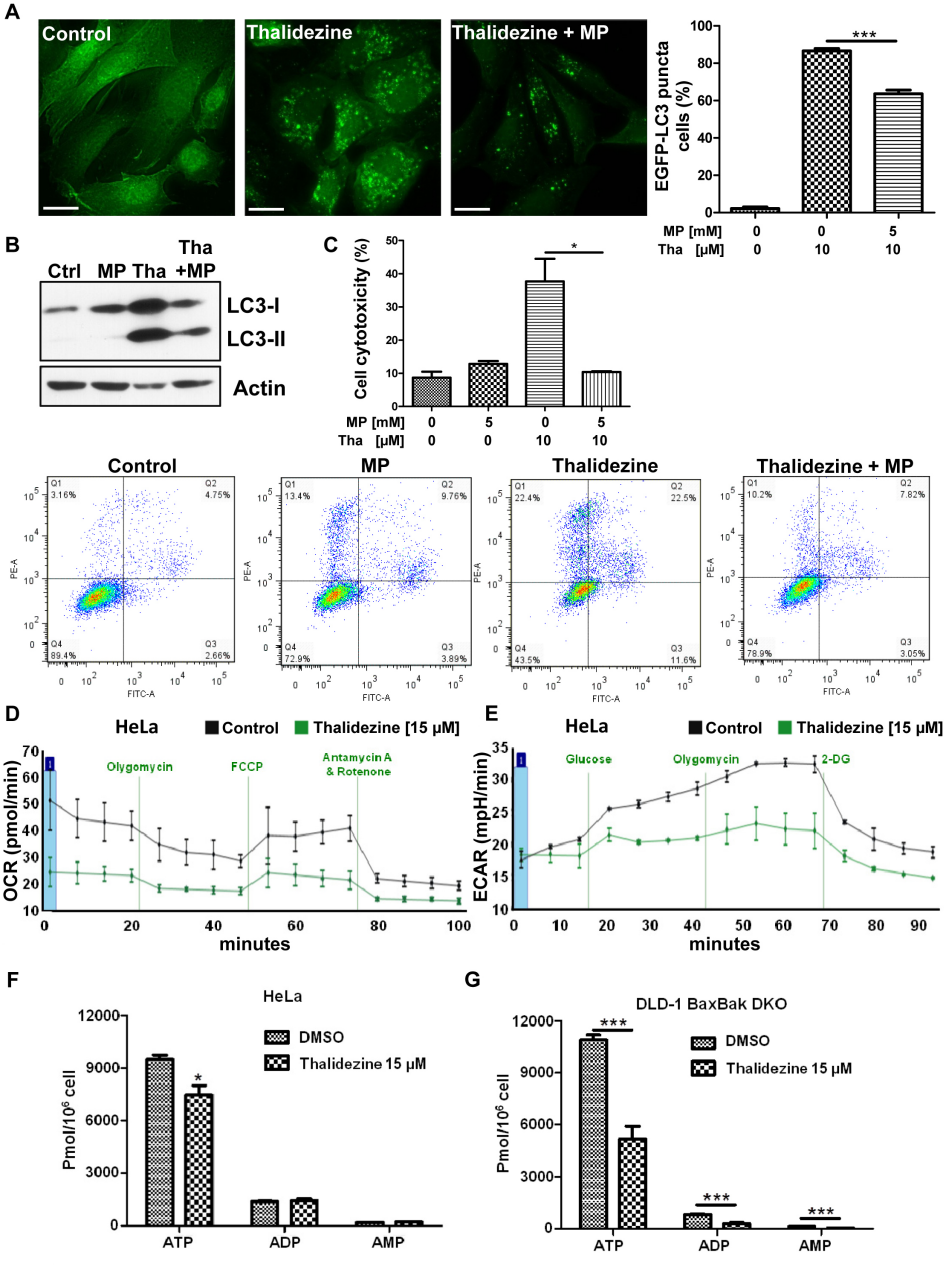
Thalidezine suppresses cancer cell energy metabolism **(A)** Methyl pyruvate suppresses the thalidezine-mediated autophagic effect in cancer cells. HeLa cells were treated with 10 μM of thalidezine (Tha) with or without 5 mM of methyl pyruvate (MP) for 24 h. Representative micrographs of autophagic cells and cell percentage with EGFP-LC3 puncta formation quantification. Scale bar = 10 μm, 60X. **(B)** Methyl pyruvate abolishes the thalidezine-mediated LC3-II conversion. Immunoblot for LC3-I, LC3-II, and β-actin detection (Uncropped blots images, Supplementary Figure 4B). **(C)** Methyl pyruvate abrogates thalidezine-mediated cell death. Annexin V stain flow cytometry analysis (lower panel) and percentage of cell death quantification (upper panel). **(D)** Thalidezine inhibits mitochondrial respiration and oxygen consumption rate (OCR). **(E)** Thalidezine inhibits glycolysis and extracellular acidification rate (ECAR). HeLa cells were treated with 15 μM of thalidezine for 24 h and then subjected to seahorse analysis using OCR and ECAR assay kits. **(F** & **G)** Thalidezine decreases the ATP production in HeLa and DLD-1 *BAX-BAK* DKO colon cancer cells. *, *P* ≤ 0.05; **, *P* ≤ 0.01; ***, *P* ≤ 0.001. Data were mean value ± S.D of three independent experiments.

AMPK was originally defined as the upstream kinase for critical metabolic enzymes implicated in lipid and glucose metabolism [1]. The two major energy pathways of cells are mitochondrial respiration and glycolysis, and the branch point of both pathways is pyruvate. To demonstrate if thalidezine autophagy induction through AMPK activation is involved in the suppression of energy production, studies using pyruvate were first performed. The glycolytic intermediate (methyl pyruvate) was able to suppress thalidezine-mediated EGFP-LC3 puncta formation and LC3-II conversion (Figure 4A & 4B), suggesting that thalidezine-induced autophagy involved energy depletion. To address whether thalidezine-mediated cell death is related to energy depletion, cytotoxicity in the presence of methyl pyruvate was evaluated using annexin V stain flow cytometry analysis. As shown in Figure 4C, thalidezine could significantly induce cell death in HeLa cancer cells, whereas the addition of methyl pyruvate completely abrogated the compound-mediated cell death. Based on these results, the effect of thalidezine in oxidative phosphorylation and glycolysis were further investigated in HeLa cells. As illustrated in Figure 4D, thalidezine treatment inhibited the mitochondrial respiration rates. HeLa cells exhibited the expected oxygen consumption rate (OCR) response to successive treatment with oligomycin, (FCCP), antimycin A and rotenone, which are well-defined small-molecule modulators of the electron transport chain. The cellular basal respiration, ATP production (after the inhibition of the ATP synthase by olygomycin), maximal respiration (after the stimulation of the OCR by FCCP), and non-mitochondrial respiration (after the mitochondrial respiration shuts down by the combination of rotenone and antimycin A) were reduced in thalidezine-treated HeLa cells. Glycolysis was reduced to some extent (Figure 4E), cells were incubated in the glycolysis stress test medium without glucose or pyruvate before the extracellular acidification rate (ECAR) measurement. ECAR was significantly higher in control cells than thalidezine-treated cells after injection of glucose and olygomycin, indicating the inhibition of glycolysis and glycolytic capacity by thalidezine treatment. These results indicated that thalidezine has inhibitory effect on mitochondrial oxidative respiratory reaction chain and glycolysis. Concomitantly, the amount of energy production in the form of ATP was significantly suppressed in HeLa cells (Figure 4F) and DLD1 *BAX-BAK* DKO apoptosis-resistant colon cancer in response to thalidezine treatment (Figure 4G). These findings suggested that thalidezine is a potent metabolic suppressor *via* AMPK activation in our cellular models. Since, the activation of AMPK which shifted the energy generation process from glycolysis to mitochondrial oxidative phosphorylation [17, 35, 36], the extent of glycolysis is reduced as demonstrated in the ECAR analysis. However, the OCR of our cancer cells was also decreased as the thalidezine-induced autophagy could remove mitochondria. Provided that the mitochondria function of most cancers is defected according to Warburg effect [37–39], the autophagic clearance of mitochondria would lead to significant changes of OCR.

### Thalidezine induces cell death via autophagy induction

**Figure 5:**
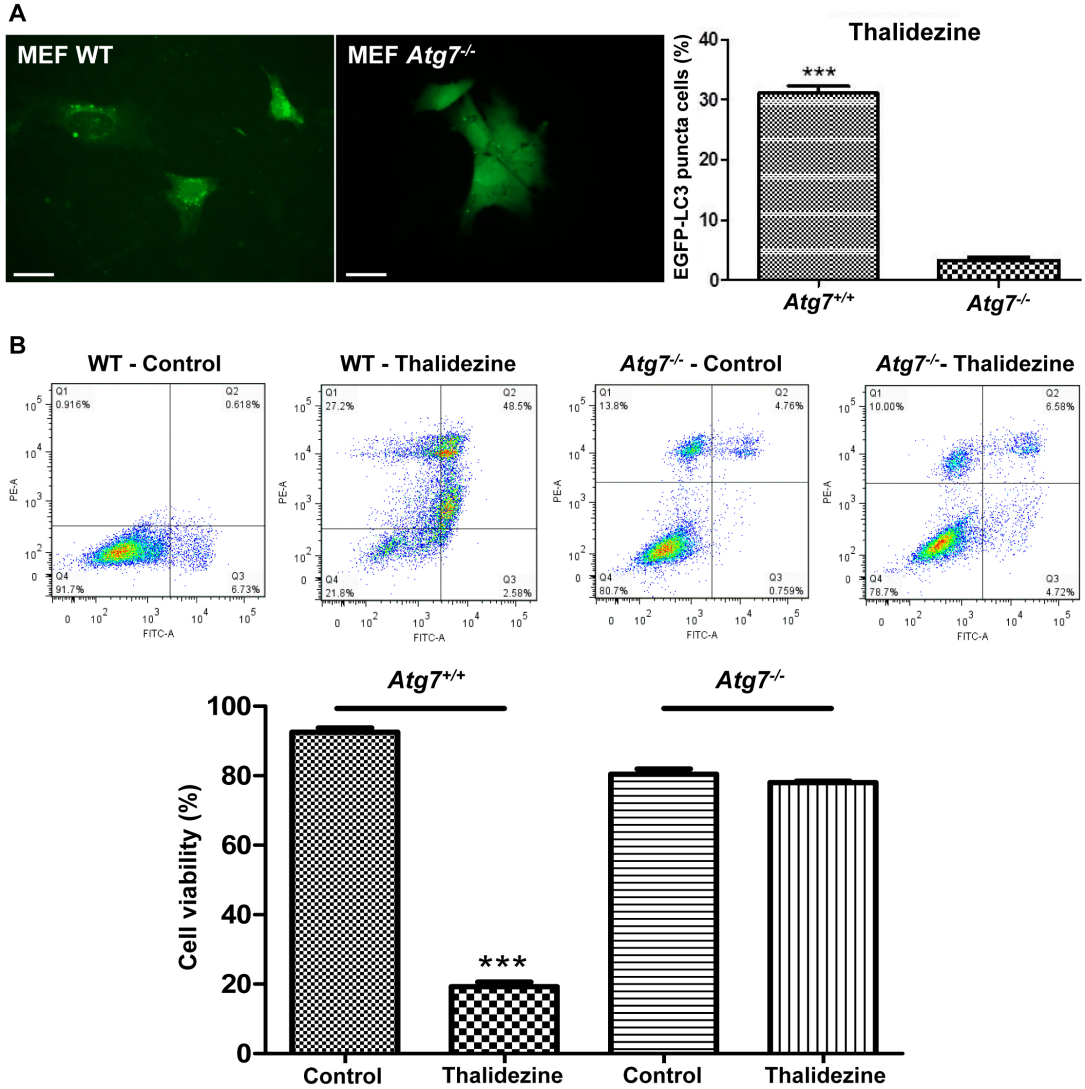
Thalidezine induces autophagic cell death in *Atg7*-dependent manner **(A)** Thalidezine induces autophagy EGFP-LC3 puncta in Atg7^+/+^ WT MEF cells. Both *Atg7^+/+^* WT and *Atg7^−/−^* deficient MEF cells were treated with DMSO (Control) or 10 μM of thalidezine (Tha) for 24 h prior to immunofluorescence imaging. Scale bar = 10 μm, 60X. Bar chart quantification of cells with EGFP-LC3 puncta formation is shown in the right panel. **(B)** Thalidezine induces cell death in *Atg7^+/+^* WT MEF cells. Annexin V stain flow cytometry analysis (upper panel) and percentage of cell viability quantification (lower panel). ***, *P* ≤ 0.001. Data were mean value ± S.D of three independent experiments.

Autophagy-related gene 7 (*Atg7*) is one of the essential genes for vesicle nucleation and elongation during autophagy induction [40]. Besides, cancer cells lacking *Atg7* gene are insensitive to response to the compounds-induced autophagy [29, 41, 42]. To examine whether thalidezine requires *Atg7* for autophagy induction, the WT and *Atg7^−/−^* MEF cells transfected with EGFP-LC3 plasmid were incubated with thalidezine for 24 h and analyzed for EGFP-LC3 puncta formation. As shown in Figure 5A, 10 μM of thalidezine significantly induced formation of EGFP-LC3 puncta in WT, but not in *Atg7^−/−^* MEF cells, indicating the involvement of *Atg7* in thalidezine-mediated autophagy induction. To address whether thalidezine-mediated autophagy induction is related to cell death, cytotoxicity in these cell lines were evaluated using annexin V stain flow cytometry analysis. Thalidezine exhibited less toxicity in *Atg7^−/−^* MEF cells when compared to their WT counterparts (Figure 5B). These data suggested that thalidezine-mediated autophagy would eventually contribute to autophagic cell death, as the failure in the induction of autophagy in *Atg7^−/−^* cells completely abolished the thalidezine-mediated cytotoxicity. Collectively, our findings suggested that thalidezine-induced autophagy requires *Atg7* and it promotes autophagic cell death in cancer cells.

### Thalidezine eliminates apoptosis-resistant cancer cells via AMPK-activated autophagic cell death

**Figure 6:**
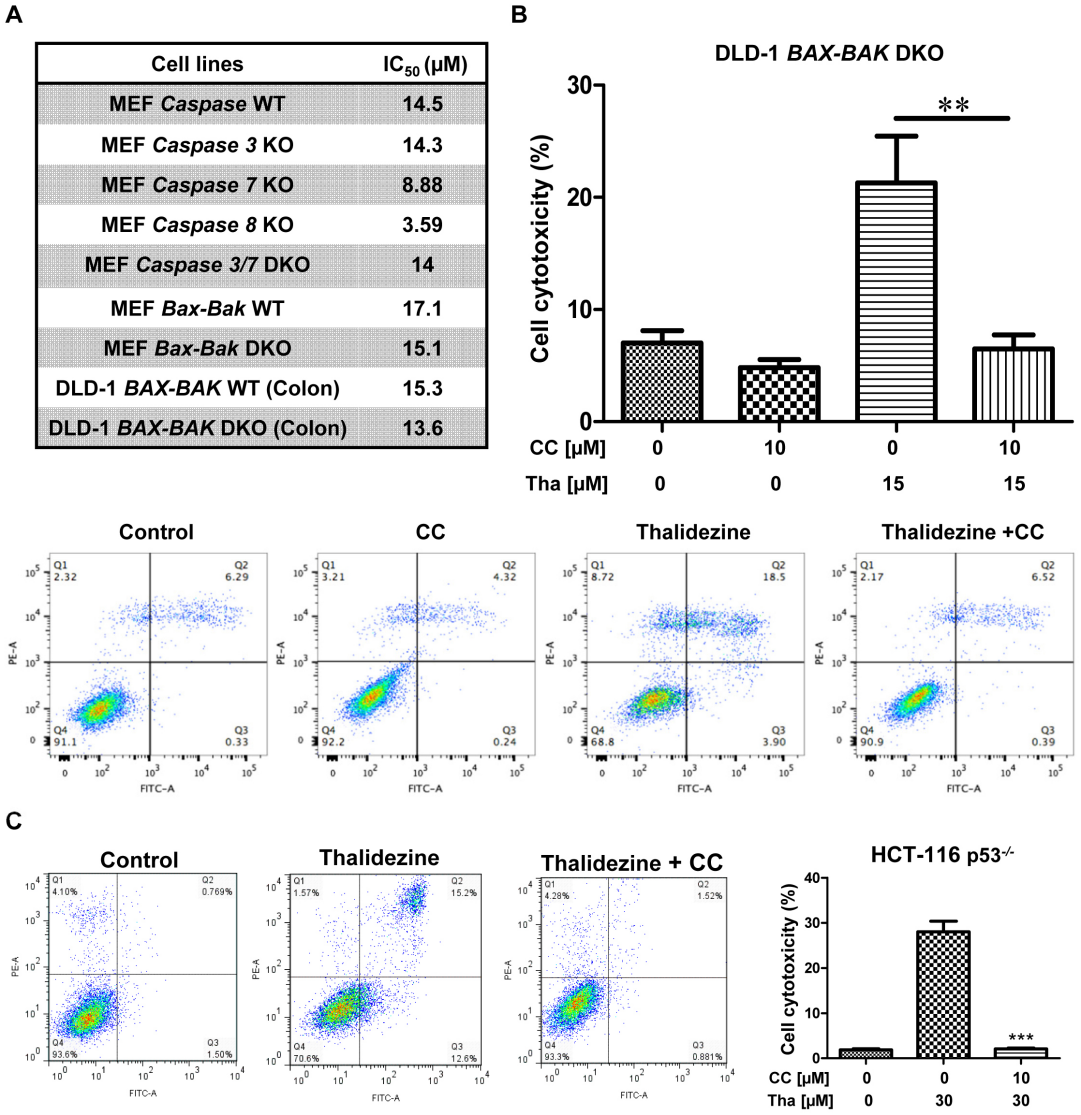
Thalidezine induces autophagic cell death in apoptosis-resistant cancer *via* AMPK signaling **(A)** Cytotoxicity of thalidezine in a panel of apoptosis-resistant cells. WT MEFs, *caspase 3*, *7*, and *8* KO, *caspase 3/7* DKO, *Bax-Bak* WT, and *Bax-Bak* DKO deficient MEFs; DLD-1 WT, and DLD-1 *BAX-BAK* DKO colon cancer cells were treated with 0-100 μM of thalidezine (Tha) for 3 days and then subjected to MTT cytotoxicity assay. **(B)** AMPK inhibitor CC abrogates thalidezine-induced autophagic cell death in DLD-1 *BAX-BAK* DKO colon cancer cells. Cells were treated with DMSO (Control) or 15 μM of thalidezine with or without 10 μM of CC for 24 h. Annexin V stain flow cytometry analysis (lower panel). Percentage of cell death quantification (upper panel). **(C)** AMPK inhibitor CC suppresses thalidezine-mediated autophagic cell death in apoptosis-resistant p53^−/−^ HCT-116 colon cancer cells. Cells were treated with DMSO or 30 μM of thalidezine with or without 10 μM of CC for 24 h. Annexin V stain flow cytometry analysis (left panel). Percentage of cell death quantification (right panel). **, *P* ≤ 0.01; ***, *P* ≤ 0.001. Data were mean value ± S.D of three independent experiments.

Cancer cells are frequently resistant to chemotherapeutic-mediated apoptosis [43]. Thus, the use of small-molecules to induce autophagic cell death in apoptosis-defective or apoptosis-resistant cancer cells could be a good strategy to overcome the problems of drug resistance [25, 44]. To investigate whether thalidezine exhibits potent cytotoxic effects towards apoptosis-resistant cells, a panel of apoptosis-defective MEF cells (*caspase 3*, *7*, *8* KO, *3/8* DKO, and *Bax-Bak* DKO) and DLD-1 *BAX-BAK* DKO colon cancer cells were used. As shown in Figure 6A, thalidezine showed similar cytotoxicity in both WT and *caspase* deficient MEF cells, and was more susceptible to induce cell death in *caspase 7* and *8 KO MEFs*. Similar cytotoxic effect towards both WT and *Bax-Bak* DKO MEF cells, and DLD-1 WT and *BAX-BAK* DKO cancer cells suggested that, thalidezine could overcome the apoptosis-resistant phenotype of cells conferred by genetic deficiencies. To further validate the above observation, cytotoxic effects of thalidezine in both WT and *Bax-Bak* DKO MEF cells were determined using annexin V stain flow cytometry analysis (Supplementary Figure 3A). As expected, both MTT and flow cytometry results were coherent to each other, suggesting that thalidezine could induce potent cytotoxicity in apoptosis-defective or apoptosis-resistant cells. Owing to the direct interaction of AMPK by thalidezine, we determined the role of AMPK in thalidezine-mediated autophagic cell death in DLD-1 *BAX-BAK* DKO apoptosis-deficient colon cancer cells. Apparently, AMPK inhibitor CC significantly suppressed the thalidezine-induced autophagy and cell death in DLD-1 cells *BAX-BAK* DKO (Supplementary Figure 3B and Figure 6B), confirming the crucial role of AMPK signalling in thalidezine-autophagic cell death in apoptosis defective cancer cells. Furthermore, another apoptosis-resistant cancer model was also utilized to evaluate the potential anti-cancer effect of thalidezine. For this purpose, apoptosis-resistant HCT-116 p53 mutant colon cancer cells were incubated with 30 μM of thalidezine in the presence of CC prior to annexin V stain flow cytometry analysis. Obviously, addition of CC markedly blocked the thalidezine-mediated cell death in these apoptosis-resistant cancer cells (Figure 6C). Concomitantly, both DLD-1 *BAX-BAK* DKO and HCT-116 p53 mutant colon cancer were found to have AMPK activation in response to thalidezine treatment (Supplementary Figure 5). Therefore, our results highlight the therapeutic potential of developing thalidezine to target apoptosis-resistant cancer via AMPK activation.

## DISCUSSION

AMPK is a sensor of cellular energy status that inhibits cell growth and proliferation, and also promotes catabolic metabolism and autophagy [1], which functions as drug target for metabolic derangements such as type 2 diabetes, cancer, and inflammatory disease [45, 46]. Despite the complex role of AMPK in cancer which functioned contextually as both tumor suppressor and promoter [47], increasing number of studies are showing the benefits of AMPK activation in the inhibition of cancer cells proliferation, migration, invasion, metabolic reprograming, and the reversion of epithelial-mesenchymal transition [14, 48–51]. Over the past years, more than 100 different natural products have been shown to activate AMPK [17], with a significant amount of them classed as polyphenols, flavonoids, terpenes, saponins, chalcones, benzoquinones, and thiazolidinediones. Our research group has previously proposed a new class of compound exhibiting direct activation of AMPK, the bisbenzylisoquinoline alkaloid [29, 30]. In this report, we have further demonstrated that thalidezine directly binds to and activates the α1β1γ1 isoform of recombinant human AMPK. We have also confirmed that the thalidezine-induced AMPK activation triggered autophagy is independent of cell type specificity. However, thalidezine showed specific cytotoxic effect towards cancer cells and exhibit low cytotoxicity in normal cells. Consequently, thalidezine could be used as anticancer or in combination with chemotherapeutics agents in a spectrum of cancers of different origins. Therefore, in-depth studies concerning the specific site of interaction between thalidezine and AMPK are required. In particular, the application of thalidezine in cancers demonstrating multidrug resistant phenotype, the main factor impeding the chemotherapy efficacy, should be emphasized. Multiple evidences are suggesting the possible role of AMPK activation in overcoming multiple drug resistance via alternation of intracellular AMP/ATP ratio [52] and molecular manipulation of the anti-cancer drugs efflux pump, P-glycoprotein (P-gp) encoded by multidrug resistance gene (MDR1) [53]. The levels of p-AMPK were found reduced in 5-FU-resistant gastric carcinoma cells. Consistently, AMPK activator (AICAR) promoted apoptosis with increased phosphorylation of AMPK, and enhanced the sensitivity of gastric cancer cells to 5-FU with reduced expression of MDR1 [54]. 2-arylthiazolidine-4-carboxylic acid amide (ATCAA)-10 is an effective cytotoxic agent that was able to dephosphorylate Akt, and phosphorylate the AMPK in A549 and HeLa cells. Besides, ATCAA are effective cytotoxic agents towards prostate, melanoma and P-gp over-expressing MES/SA/Dx5 multidrug resistant cells [52]. Therefore, targeting the phosphatidylinositol-3-kinase (PI3K)/Akt and AMPK pathways may be effective in anti-cancer therapy. In fact, PI3K/AMPK/AKT and MAPK pathways are therapeutic targets for non-small cell lung cancer (NSCLC). 21α-Methylmelianodiol (21α-MMD), an active triterpenoid isolated from *Poncirus trifoliate*, regulates the PI3K/AKT/AMPK and MAPK pathways, and reverses the MDR activity through the inhibited expressions of P-gp or MDR1, suggesting its anti-lung cancer property through reversal of MDR activity [55]. MDR is one of the major barriers for breast cancer treatments. Emerging researches have suggested the effect of metformin on reversing chemo-resistance in breast cancer cells. For example, metformin resensitized multidrug-resistant MCF7/5-FU and MDA-MB-231 breast cancer cells to toxic chemotherapy agents including 5-fluorouracil (5-FU), adriamycin, and paclitaxel, together with the activation of AMPK signal pathway [56]. Targeting the B-Raf kinase in the AMPK signaling pathway is one of the therapeutic approaches in cancer therapy. Although two Raf inhibitors, sorafenib and PLX4720, were demonstrated to inhibit the growth of MDR-NIH 3T3 cells with different phosphorylation extent on AMPK, both of them inhibited mTOR and induced autophagy, suggesting the possible role of AMPK activation and Raf inhibition in abrogating the multidrug resistance of cancers [57]. Mollugin, a compound isolated from roots of *Rubica cordifolia L*., inhibited transcription and expression of MDR1 through attenuating CRE transcriptional activity via AMPK activation [58].

Although AMPK-induced autophagy appeared to be a promising pharmaceutical target for drugs against malignancy, technicalities rendering the practical use of AMPK modulators are still an issue for successful clinical translation. For example, the complex genetic heterogeneity within tumor mass implied the difficulties in accurately applying the compatible AMPK activators which is relevant to the specific subclonal mutations [59]. In the tissue and organ levels, autophagy is highly cell-type specific which may reduce the efficacy of the applied AMPK activators. As described in chronic liver disease, hepatic fibrosis, and hepatocellular carcinoma, the coexistence of hepatocytes and hepatic stellate cells (HSCs) within the hepatic lobes could critically affect the therapeutic performance in response to the different stages of disease progression [19]. Also, the timing of autophagy activators administration is another factor that needs to be fully examined, since the nutrients that generated from autophagy induction may provide energy for supporting cancer development upon some stages of tumorigenesis [31].

Metformin is an AMPK activator that acts indirectly by inhibiting complex I of the respiratory chain, while we showed that thalidezine directly activated AMPK. As a result, the therapeutic effects of using thalidezine could be more specific than metformin. In addition, the use of metformin is associated with the side effect of lactic acidosis [60]. When compared, thalidezine is isolated from the natural medicinal plant *Thalictrum glandulosissimum*, which have long been used in traditional Chinese medicine therapy. Therefore, thalidezine could be safer than the synthetic compound metformin. Furthermore, thalidezine can induce direct AMPK activation and the downstream autophagic process. The compound also triggers cytotoxic effects upon cancer without causing significant cell death of normal cell. Such cytotoxicity is associated with autophagy, making thalidezine an ideal compound to be further developed into the novel and safe AMPK activator effective for multidrug resistant cancers with hampered apoptotic response. Notably, our data suggested that thalidezine is an energy suppressor by acting through AMPK-mediated metabolic inhibition, therefore, the compound could also be useful treatment for metabolic diseases including diabetes and inflammation. Such finding also encouraged the search of other innovative pharmaceutical candidates for multidrug resistant cancers by targeting the energetic metabolic pathways.

## MATERIALS AND METHODS

### Cell culture

HeLa, MCF-7, PC3, Hep3B, HepG2, A549, H1975, H1299, LO2 cell lines were purchased from the American Type Culture Collection (Rockville, MD). DLD-1, and DLD-1 *BAX/BAK* double knockout (DKO) cell lines were from Sigma-Aldrich. These cell lines were authenticated by ATCC. Immortalised wild type (WT) and *Atg7*-deficient mouse embryonic fibroblasts (MEF) were obtained from Prof. Masaaki Komatsu (Juntendo University, School of Medicine, Japan). Immortalised WT, Caspase 3, 7, knockout (KO), and caspase 3/7 DKO MEF were graciously provided by Prof. Richard A. Flavell (Yale University School of Medicine, United States). Immortalised WT and Caspase 8 KO MEF were a gift from Prof. Kazuhiro Sakamaki (Kyoto University, Graduate School of Biostudies, Japan). Immortalised WT and *Bax-Bak* DKO MEF were gently supplied by Prof. Shigeomi Shimizu (Tokyo Medical and Dental University, Medical Research Institute, Japan). HCT-116 WT and p53^−/−^ deficient colon cancer cell lines were kindly provided by Professor Bert Vogelstein (Ludwig Center at Johns Hopkins, Howard Hughes Medical Institute, USA). Every culture medium was supplemented with 10% foetal bovine serum (FBS), 50 U/mL penicillin, and 50 μg/mL streptomycin (Invitrogen, Scotland, UK). Cells were cultured at 37°C in a humidified incubator containing 5% CO_2_.

### Reagents, plasmids and antibodies

The following reagents were used at doses indicated in the text and figures. Thalidezine (China Chengdu Biotechnology Company Ltd., Chengdu, China) (>98% purity, HPLC). Bafilomycin A, E64D, pepstatin A, and compound C (Calbiochem, Darmstadt, Germany). The pGFP-LC3 and mRFP-GFP tandem fluorescent-tagged LC3 (tfLC3) plasmids were kindly provided by Prof. Tamotsu Yoshimori (Osaka University, Japan). Antibodies against LC3-I, LC3-II, p-AMPK (Thr172), AMPK, p-p70S6K (Thr389), and p70S6K were ordered from Cell Signalling Technologies Inc. (Beverly, MA). The ZyMax™ TRITC-conjugated anti-mouse secondary antibodies were purchased from Invitrogen (Scotland, UK). Actin antibody was obtained from Santa Cruz Biotechnology (Santa Cruz, CA). Unless otherwise specified, all other reagents were purchased from Sigma-Aldrich (MO, USA).

### Bio-Layer Interferometry (BLI) binding assay

The binding kinetics of thalidezine to recombinant active human AMPK (Sigma-Aldrich, St. Louis, USA) was determined using BLI on Octet RED (FortéBio, Shanghai, China) following manufacturer protocol. All the interaction analyses were performed at 30°C in PBS 0.2% DMSO buffer. Loading of Nickel-charged tris-NTA (Ni-NTA) biosensors (FortéBio) was conducted by exposing HIS-tagged AMPK (α1/β1/γ1, Catalog Number A1233) containing 0.1 mg/mL to biosensor tips for two hours. The 96-well microplates used in the Octet were filled with 200 μl of sample or buffer per well and agitated at 1000 rpm. The loaded biosensors were washed in buffer for 600 sec and transferred to the wells containing thalidezine at concentrations of 12.5, 25, 50, and 100 μM in buffer, respectively. The association and dissociation was observed for 60 sec for each sample diluents. Reference measurements were conducted by using buffer instead of thalidezine. A parallel set of Ni-NTA unloaded biosensors was prepared to act as a control. The sample-sensorgrams were corrected by subtracting the double reference curve. Kinetic parameters (*Kon* and *Koff*) and affinity (*K_D_*) were determined from a global fit to a 1:1 binding model of the data between AMPK and thalidezine using Octet software (FortéBio).

### AMPK kinase activity

AMPK kinase activity was measured by CycLex® AMPK Kinase Assay Kit (MBL, Japan) according to manufacturing instructions. Recombinant active human AMPK (α1 β1 γ1) was incubated with the indicated concentrations of thalidezine or AMP (10 μM) for 20 min at 30°C in a plate pre-coated with the protein mouse insulin receptor substrate-1 (IRS-1). After stop the reaction by washing five times, AMPK activity was measured using an anti-mouse phospho-Ser-789, and peroxidase-coupled anti-mouse IgG antibody (30 minutes at RT). Finally, conversion of the chromogenic substrate tetra-methylbenzidine was quantified by measuring changes in absorbance at 450/550 nm.

### Immunoblot analysis

Western blot analysis was carried out following standard methods. Cells were lysed with RIPA lysis buffer with protease and phosphatase inhibitor cocktails. Protein concentrations were determined using the Bio-Rad protein assay (Bio-Rad Laboratories, Inc., Hercules, CA, USA). After electrophoresis, the proteins from SDS/PAGE were electro-transferred to a Hybond enhanced chemiluminescence nitrocellulose membrane (Amersham Biosciences, NJ, USA), which was then blocked with 5% dried milk for 1 hour. After washing, the blot was incubated with the indicated primary antibodies overnight at 4°C. Detection was performed using appropriated HRP-conjugated secondary antibodies for 1 hour at RT followed by chemiluminescence (Invitrogen). Band intensities were quantified by using the software ImageJ (NIH, MD, USA). LC3 conversion was quantified by measuring band intensities (LC3-II, 16 kDa) and normalised to β-actin.

### MTT cytotoxicity assays

Cytotoxicity of thalidezine was measured using the MTT (3-[4,5-dimethylthiazol-2-yl]-2,5 diphenyl tetrazolium bromide) assay in six replicates. Cells were seeded and incubated in 96-well plates in the respective medium, and then exposed to various concentrations of thalidezine dissolved in DMSO for 72 hours. After MTT incubation, the absorbance at 570 nm was determined on a plate reader. The percentage of viable cells was calculated using the following formula: Cell viability (%) = Cells number _treated_/Cells number _DMSO control_ × 100.

### Quantification of autophagic EGFP-LC3 puncta

Cells transiently transfected with the EGFP-LC3 plasmid were treated with DMSO or thalidezine. The samples were fixed with 4% paraformaldehyde (Sigma-Aldrich) and then mounted with FluorSave™ Reagent (Calbiochem, San Diego, California). GFP positive cells were imaged by widefield epifluorescence microscopy using Photometrics CoolSNAP HQ2 CCD camera on the Olympus IX71-Applied Precision DeltaVision restoration microscope (Applied Precision, Inc, USA). All fluorescence images were deconvolved using DeltaVision algorithms (Applied Precision, Inc.). To quantify autophagic cells, the percentage of cells with increased EGFP-LC3 puncta formation was calculated by counting the number of cells showing the punctate pattern of EGFP-LC3 (≥ 10 puncta/cell) divided by the total number of EGFP-positive cells. At least 1000 cells from randomly selected fields were scored per condition and experiment.

### mRFP-GFP tandem fluorescent-tagged LC3 (tfLC3) detection

HeLa cancer cells transfected with tfLC3 were treated with thalidezine, and processed as described above. Colocalisation of mRFP (red) with GFP (green) fluorescence protein in tfLC3 puncta was measured using DeltaVision algorithms (Applied Precision, Inc.), and shown as the percentage of the total number of yellow mRFP^+^-GFP^+^ puncta. At least five images selected fields were scored per condition and experiment.

### Endogenous LC3-II immunofluorescence analysis

Cells were fixed with 4% paraformaldehyde, permeabilized with methanol, incubated with anti-LC3II [1:200] in TBST 5% BSA overnight at 4°C, incubated with TRITC anti-mouse secondary antibody [1:1000] in TBST 5% BSA at 37°C for 1 h in darkness, mounted, and imaged by epifluorescence microscope.

### Flow cytometry analysis

Thalidezine-treated cells were harvested and analysed by multiparametric flow cytometry using FITC-Annexin V and propidium iodide staining (BD Biosciences, San Jose, CA, USA) according to the manufacturer instructions. Apoptotic cells were quantitatively counted by a flow cytometer (BD FACSAria III, San Jose, CA, USA). Data acquisition and analysis were performed with CellQuest (BD Biosciences, San Jose, CA, USA) from triple independent experiments.

### Metabolic stress assay

HeLa cells were plated in XFp plates (SeaHorse Biosciences) at 3000-5000 cells/well, wells without cells was added as background control. After 24 hours of thalidezine treatment at 15 μM, oxidative phosphorylation and glycolysis were determined in a SeaHorse Bioscience XFp extracellular flux analyzer according to manufacturer instructions. For the mitochondrial oxygen consumption rate (OCR), the compounds oligomycin (10 μM), Carbonyl cyanide 4-(trifluoromethoxy) phenylhydrazone (FCCP) (0.5 μM), and a mix of rotenone (complex I inhibitor) and antimycin A (complex III inhibitor) (0.5 μM) were serially injected to measure ATP production, maximal respiration, and non-mitochondrial respiration, respectively. Oligomycin reduces OCR by inhibiting ATP synthase (complex V) which decreases the mitochondrial respiration associated with cellular ATP production. FCCP collapses the proton gradient and disrupts the mitochondrial membrane potential which maximizes the oxygen consumption by complex IV. The mix prepared from rotenone and antimycin A switch off mitochondrial respiration and enable the calculation driven by processes outside the mitochondria. While for the extracellular acidification rate (ECAR), the compounds glucose (10 mM), oligomycin (10 μM), and 2-deoxy-glucose (2-DG, 50 mM) were serially injected to measure glycolysis, glycolytic capacity, and non-glycolytic acidification. The first injection is to saturate the glucose concentration which facilitates the glycolytic pathway for ATP and protons synthesis resulting in a rapid increase in ECAR. Oligomycin shifts the energy production to glycolysis by inhibiting the mitochondrial ATP production. The glucose analog, 2-deoxy-glucose, inhibits glycolysis through competitive binding to glucose hexokinase.

### LC-MS/MS measurement of ATP metabolites

The HeLa cell pellet was then treated with 150 μL of 15% trichloroacetic acid (TCA) containing 7.5 μL of 20.0 uM ATP13C, 15N as internal standard and placed on ice for 10 minutes. After centrifugation at 13,500 rpm for 15 min, the acidic supernatant was separated and neutralized twice with 80 μL mixture of trioctylamine and 1, 1, 2-trichlorotrifluoroethane (a volume ratio of 45 to 55) for LC-MS/MS analysis. A Thermo Fisher TSQ LC–MS/MS system consisted of an Accela Autosampler, an Accela pump and a Quantum Access triple quadrupole mass spectrometer. Data acquisition was performed with the Xcalibur software version 2.0.7, and data processing was carried out using the Thermo LCquan 2.5.6 data analysis program.

### Statistical analysis

The results were expressed as the means ± SD as indicated. All statistical analyses were performed using a two-tailed Student t test. P < 0.05 was considered statistically significant.

## SUPPLEMENTARY MATERIALS FIGURES AND TABLES



## Abbreviations

AMPK: AMP-activated protein kinase
ULK: Protein kinases that initiate autophagy
mTORC1: Mammalian target of rapamycin complex 1
*Atg*: Autophagic genes
AICAR: 5-aminoimidazole-4-carboxamide-1-b-d-ribofuranoside
CHM: Chinese herbal medicines
P-gp: P-glycoprotein
MDR: Multidrug resistance gene
WT: Wild type
DKO: Double knockout
MEF: Mouse embryonic fibroblasts
KO: Knockout
tfLC3: mRFP-GFP tandem fluorescent-tagged LC3
BLI: Bio-Layer Interferometry
OCR: Oxygen consumption rate
ECAR: Extracellular acidification rate
2-DG: 2-deoxy-glucose
