# Supplementary material for: Thalidezine, a novel AMPK activator, eliminates apoptosis-resistant cancer cells through energy-mediated autophagic cell death
Source: Oncotarget. 2017 Feb 22;8(18):30077–91. doi: 10.18632/oncotarget.15616 (PMC5444727; doi:10.18632/oncotarget.15616)
Supplement: Supplementary file 1 [file oncotarget-08-30077-s001.pdf]

# Thalidezine, a novel AMPK activator, eliminates apoptosis-resistant cancer cells through energy-mediated autophagic cell death

## SUPPLEMENTARY FIGURES

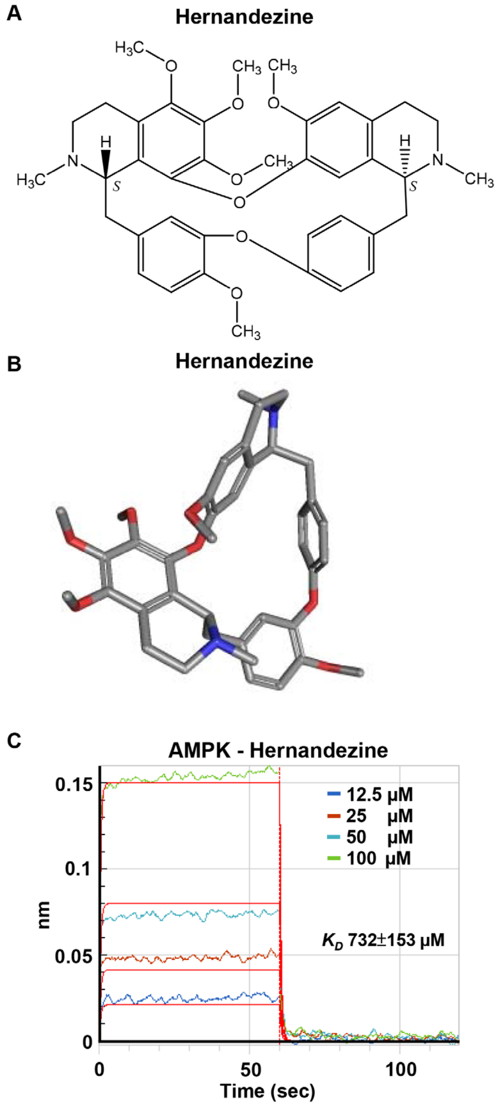


**Supplementary Figure 1: (A) Chemical structure of hernandezine. (B)** 3D conformer of hernandezine and thalidezine (blue-nitrogen, red-oxygen) **(C)** Kinetic analysis of the interaction between AMPK and hernandezine by BLI.

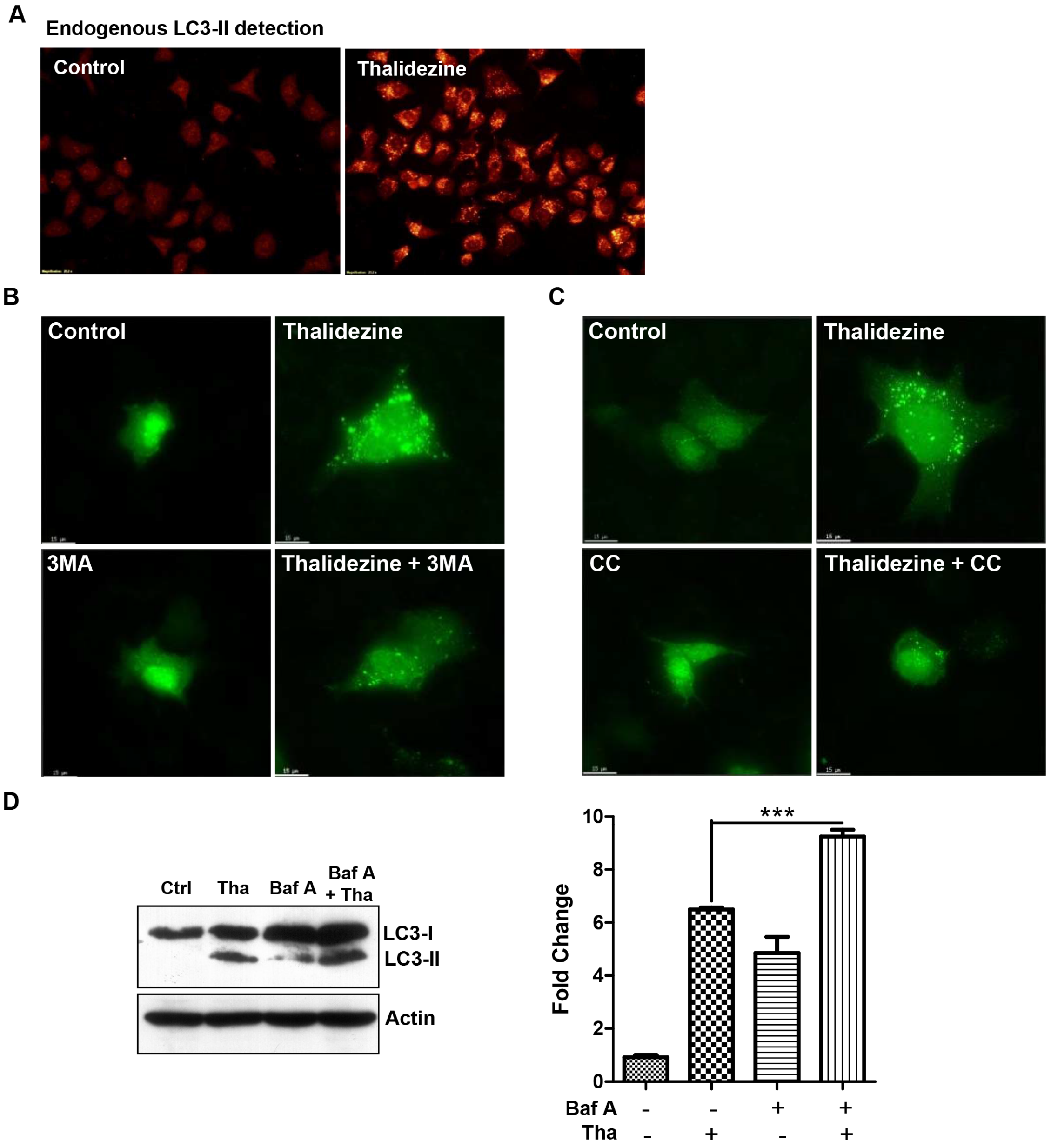


**Supplementary Figure 2: (A) Endogenous LC3-II expression in HeLa cells. (B & C)** HeLa cells transiently transfected with EGFP-LC3 plasmid were treated with DMSO (Control) or thalidezine (Tha, 10 µM) with or without 3-MA (5 mM), and with or without CC (5 µM) for 4 h. Representative fluorescence microscopy images were captured at 60X magnification. Scale bar = 15 µm. **(D)** HeLa cells were treated with 10 µM of thalidezine (Tha) in the presence or absence of 50 nM bafilomycin A (Baf A) for 8 h. Immunoblot for LC3-I, LC3-II, and actin detection (left). LC3 conversion was expressed as fold change relative to the DMSO-treated negative control (Ctrl, right). ***, $P \leq 0.001$. Data were mean value ± S.D of three independent experiments.

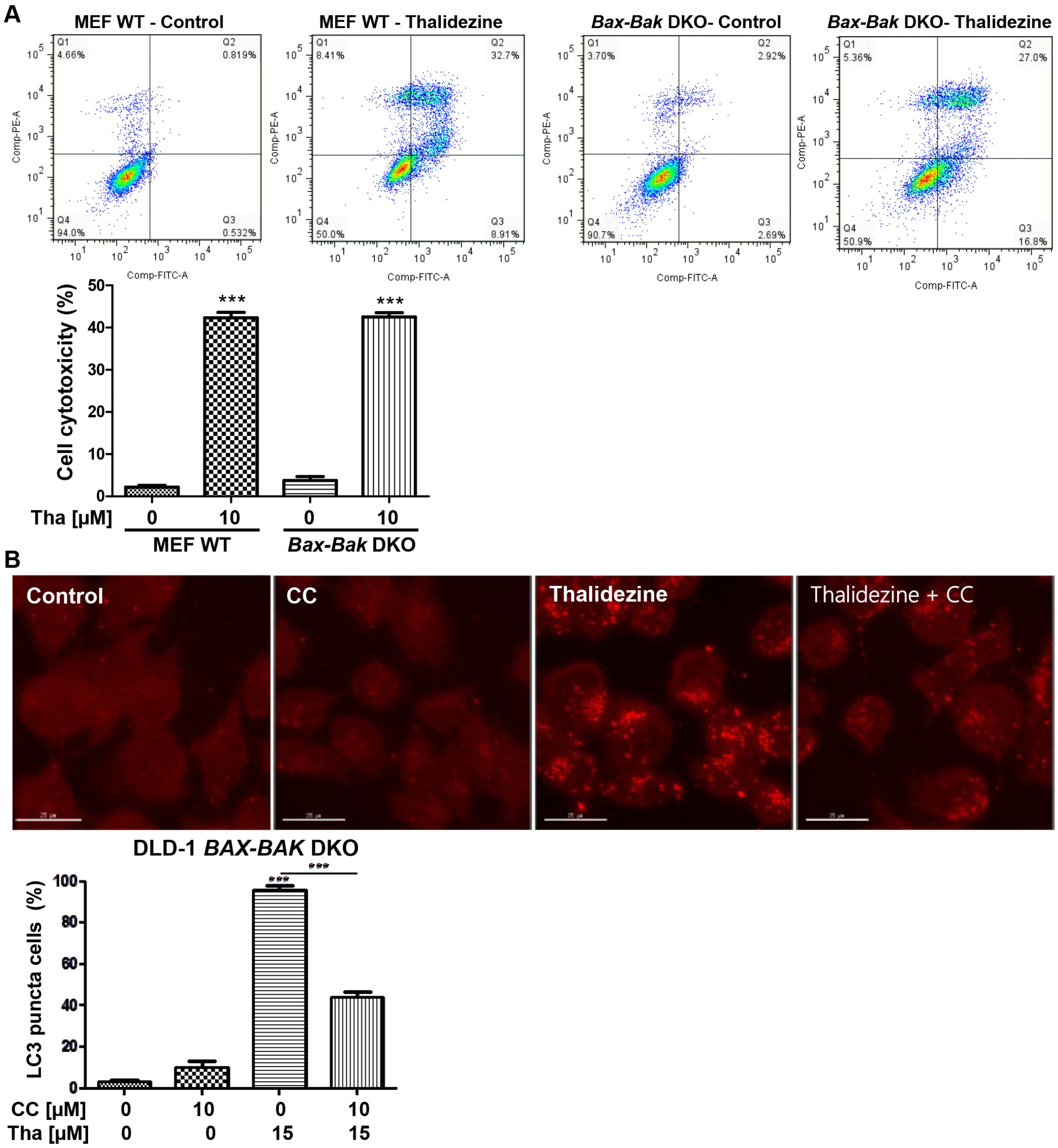


**Supplementary Figure 3: (A)** WT and *Bax-Bak* DKO deficient MEF were incubated with DMSO (Control) or 10 μM of thalidezine for 24 h. Annexin V stain flow cytometry analysis (upper). Percentage of cell death quantification (lower). **(B)** AMPK inhibitor CC abrogates thalidezine-induced autophagy in DLD-1 cells *BAX-BAK* DKO colon cancer. Cells were treated with DMSO (Control) or 15 μM of thalidezine with or without 10 μM of CC for 24 h. Endogenous expression of LC3-II (upper panel). Scale bar = 15 μm, 60X. Cell percentage with endogenous LC3-II puncta formation quantification (lower panel). ***, $P \leq 0.001$. Data were mean value ± S.D of three independent experiments.

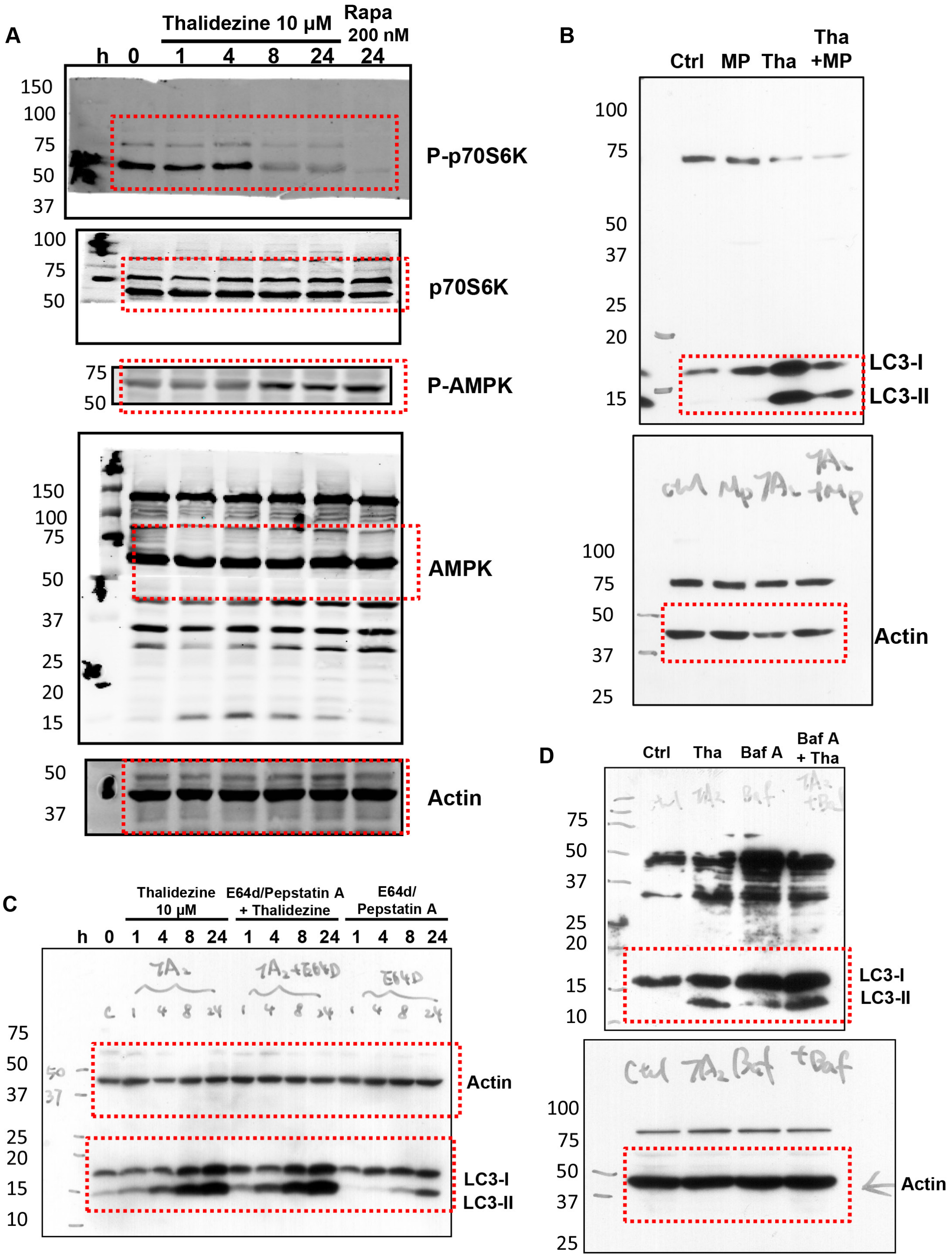


**Supplementary Figure 4: Uncropped scans of western blots included in figures.**

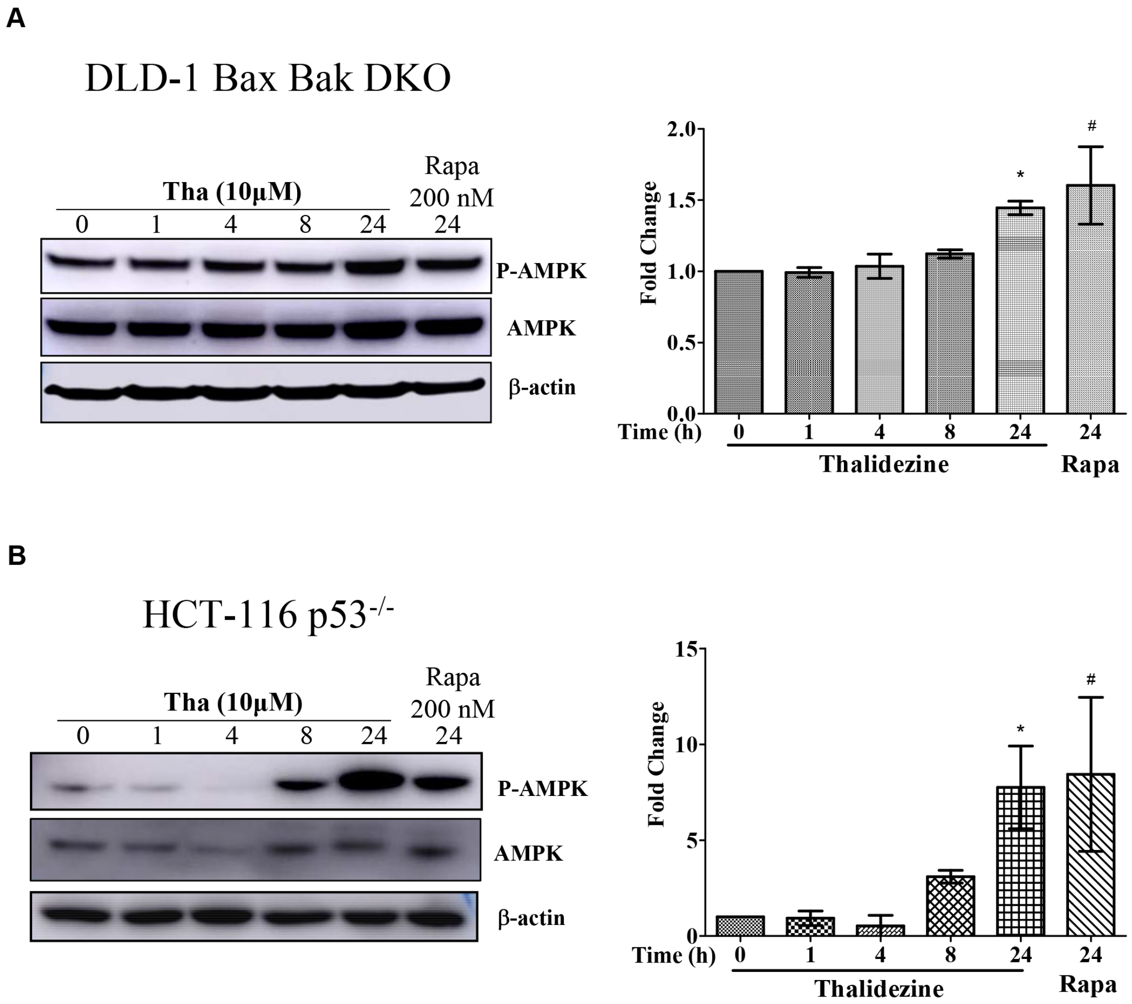


**Supplementary Figure 5: Thalidezine activates the AMPK signaling pathway. (A)** DLD-1 *BAX-BAK* DKO colon cancer cells were treated with 10 µM of thalidezine for 0-24 h, rapamycin (Rapa, 200 nM) was used as the positive control. **(B)** $p53^{-/-}$ HCT-116 colon cancer cells were treated with 10 µM of thalidezine for 0-24 h, rapamycin (Rapa, 200 nM) was used as the positive control. Immunoblots indicated p-AMPK, total AMPK and β-actin detection. Data were representative of three to five independent experiments.
